# Effects of Dietary Vitamin A on the Growth Performance, Nonspecific Immune Response, Shell Microbiota and Red Spotted Disease Resistance of Juvenile Sea Urchin (*Strongylocentrotus intermedius*)

**DOI:** 10.1155/anu/3601517

**Published:** 2025-02-07

**Authors:** Dan Gou, Rujian Xu, Haijing Liu, Panke Gong, Weixiao Di, Huinan Zuo, Jun Ding, Yaqing Chang, Rantao Zuo

**Affiliations:** Key Laboratory of Mariculture and Stock Enhancement in North China's Sea (Ministry of Agriculture and Rural Affairs), Dalian Ocean University, Dalian 116023, China

**Keywords:** composition of shell microorganisms, growth, immunity, *Strongylocentrotus intermedius*, vitamin A

## Abstract

A 114-day feeding trial was used to investigate the influence of vitamin A (VA) on growth performance, nonspecific immune responses and shell microbiota in juvenile sea urchin (*Strongylocentrotus intermedius*). Graded levels of VA (0, 4000, 8000, 16,000, 32,000 and 64,000 IU/kg) were added to make six experimental feeds. Each feed was allocated to three parallel tanks of sea urchins (initial weight 0.87 ± 0.05 g and initial test diameter 1.83 ± 0.57 mm). The data revealed that the weight gain rate (WGR) and gonadosomatic index (GSI) rose markedly as VA addition level increased from 0 to 4000 IU/kg and then reached a plateau with further increase of dietary VA levels. As VA addition level increased, nonspecific immune response of *S. intermedius* first increased and then decreased, with those fed diets with relatively higher addition of VA (32,000 IU/kg) exhibiting significantly greater phagocytic activity (PA) and acid phosphatase (ACP) activities, as well as upregulated expression of several immune-related genes such as tumour necrosis factor α (*TNF-α*), antimicrobial peptides (*AMPs*), toll-like receptors (*TLRs*) and lysozyme (*LYZ*). The abundance of Firmicutes, Bacteroidota, *Bacteroides* and *Faecalibacterium* increased, but that of Proteobacteria and *Leucothrix* decreased in the shell of *S. intermedius* as VA addition level increased. The percentage of sea urchins with severe red spotted disease decreased from 64.44% to13.33% as VA addition level increased to 32,000 IU/kg and subsequently increased to 42.22% with further increase of VA addition level. On the contrary, the percentage of sea urchins with mild red spotted disease increased from13.33% to 55.55% as VA addition level increased to 32,000 IU/kg and subsequently decreased to 31.11% with further increase of VA addition level. These results demonstrated that a low addition level of VA (4000 IU/kg) can help *S. intermedius* achieve ideal growth performance. However, relatively higher addition levels of VA (32,000 IU/kg) enhanced nonspecific immunity and red spotted disease resistance of *S. intermedius*, which could be accomplished by promoting immune gene expression and optimizing the shell microbiota composition.

## 1. Introduction

The gonads of sea urchins are highly favoured by customers around the world because of their unique texture, excellent taste and rich nutritional components [1–4]. There has been a significant decline in the natural population of edible sea urchins in recent decades due to the rising demand for this seafood [5–7]. To reduce the reliance on wild resources, attempts have been made to breed sea urchins in captivity and has subsequently achieved success in many countries and regions [8]. Originally found in the Russian Far East and off the coast of Hokkaido, Japan, the sea urchin (*Strongylocentrotus intermedius*) was brought to China in 1989 by Dalian Ocean University [1, 9]. At present, in the North Sea of China, the *S. intermedius* is becoming an important species of sea urchin used for aquaculture [10]. When the water temperature rises above 20°C, the farming of *S. intermedius* is often challenged by the occurrence of red spotted disease caused by *Vibrio* [11–13]. The symptom is first characterized by obvious external black or purple spotted lesions with dark red mucus coverage at the lesions, followed by shell ulcers, gonadal overflow and even death [14–16]. At present, the use of antibiotics is still the main means to cure red spotted disease. However, long-term use of antibiotics not only reduces their efficacy but also poses threats to the environment and food safety [13, 17]. Furthermore, it is impossible to use antibiotics in floating marine cages, an important culture pattern for *S. intermedius* [18].

Immune stimulants, such as vitamins and amino acids, have the advantages of being safe, nontoxic, cost-effective and environmentally friendly. They can be used as promising alternatives to antibiotics to prevent disease outbreaks in aquaculture [19]. Vitamin A (VA) is crucial for supporting essential physiological functions, including growth, immune response, reproduction and the development of embryos and larvae [20]. Notably, the optimal VA requirement for achieving the best immune function has been studied in several aquatic species, including tilapia (*Oreochromis niloticus*) [21], grass carp (*Ctenopharyngodon idella*) [22], juvenile Chinese mitten crab (*Eriocheir sinensis*) [23] and kuruma shrimp (*Marsupenaeus japonicus*) [24]. It was concluded that the optimal VA need was estimated to be 2000–7500 IU/kg dry diet based on several immune parameters. The immune response and disease resistance of *C. idella* can be decreased by VA deficiency [22, 25] and silver sillago (*Sillago sihama*) [26]. In contrast, excessive VA causes growth inhibition, oxidative stress, liver damage and even death in Wuchang bream (*Megalobrama amblycephala*) [20], rainbow trout (*Oncorhynchus mykiss*) [27] and juvenile *E. sinensis* [28].

The mechanisms by which VA regulates nonspecific immunity in aquatic animals (such as fish) have been explored in several studies. The expression of the immune-related genes tumour necrosis factor *α* (*TNF-α*), antimicrobial peptides (*AMPs*), toll-like receptors (*TLRs*), complement component 3 (*C3*) precursor, cysteine-aspartic protease 8 (*Caspase-8*) and lysozyme (*LYZ*) was shown to be sensitive to VA in the diet. Furthermore, VA can regulate immunity by affecting the intestinal microbiota composition [29]. For example, VA increased the relative abundance of *Akkermansia* bacteria, which enhanced the barrier function of the digestive tract and immune responses of American mink (*Neovison vison*) [30]. VA deficiency changes the Bacteroidetes/Firmicutes ratio and decreases the abundance of butyrate-producing bacteria, thereby impairing the intestinal immune function of mice (*Mus musculus*) [29]. The shell serves as a protective barrier between internal organs and water environments [31, 32]. On the shell surface (skins), the homeostatic balance of microbiota, such as bacteria, fungi and viruses, plays an important role in maintaining skin health [33]. VA can also promote host defence against skin infections by regulating the expression of AMPs and thus prevent pathogenic bacterial reproduction on the skin of *M. musculus* [34, 35]. However, according to our knowledge, the influence of dietary VA on immune-related genes expression and shell microbiota composition of *S. intermedius* have not been studied.

Therefore, the present study was undertaken to investigate the influence of dietary VA on growth performance, non-specific immune response and associated gene expression, as well as shell microbial composition of the juvenile *S. intermedius*. The purpose of present study is to confirm (1) the optimal VA addition for juvenile *S. intermedius* based on the growth performance, nonspecific immunity and red spotted disease resistance and (2) the effects of VA on shell microbiota composition and immune-related gene expression. This study is expected to provide theoretical support for optimizing the feed formulation, which is helpful for enhancing the growth and red spotted disease resistance of *S. intermedius* under intensive farming conditions.

## 2. Materials and Methods

### 2.1. Ethics Statement

The Ethics Committee of Dalian Ocean University did not require the study to be reviewed or approved because the sea urchins are invertebrates.

### 2.2. Experimental Diets and Feeding Experiment

In this experiment, the primary sources of protein were fish meal, soybean meal and wheat flour. The primary sources of lipids were fish oil and soy lecithin. VA acetate (purity ≥ 97%, Merck, Shanghai, China) was added to the six experimental feeds. The basic feed formulation was designed according to the nutritional requirements for *S. intermedius* [2, 4]. The crude protein and crude lipid of the basal feed were 21.8% and 6.13%, respectively. Based on the findings of Hassan et al. [36] and Hernandez et al. [37], it was demonstrated that the optimal VA requirement ranged from 4000 to 20,000 IU/kg for aquatic animals. Therefore, in the present study, graded levels of VA (0, 4000, 8000, 16,000, 32,000 and 64,000 IU/kg) were added to the basic diet and named A0, A4000, A8000, A16000, A32000 and A64000 (Table 1).

All solid feeds were thoroughly ground to ensure that they passed through a 60-mesh screen. First, the finely ground solid ingredients were weighed according to the proportion of the feed formulation and sequentially placed into plastic bags for mixing. Secondly, an appropriate amount of oil was weighed and mixed into the solid mixture, which was then mixed and passed through a 45-mesh sieve. Subsequently, 35% water was added and mixed well with the mixture. The prepared mixture of feed was finally subjected to extrusion and pelletization by using a machine (DES-TS1280, Dingrun, Jinan, China). After that, the feed pellets sized 2 mm × 5 mm were placed on a drying tray and dried at a temperature of 50°C. Lastly, they were cooled, packaged and stored in the refrigerator at a temperature of −20°C.

The feeding test was conducted from April 24 to August 18 at the experimental base of Dalian Ocean University (Dalian, China). Sea urchins were obtained from a farm in Lvshun (Dalian, China). The seawater was initially sand filtered prior to being utilized in the feeding experiment. The *S. intermedius* were held in a 1000 L tank and fed the formulated feeds without the extra addition of VA until they adapted to the environments and feeding regime. Then, 324 healthy *S. intermedius* deprived for 36 h were randomly distributed into 18 square plastic boxes (30 L for each box). Afterwards, the body weight, test height and test diameter of the sea urchins in each plastic box were individually measured. Each feed was randomly assigned to three plastic boxes of sea urchins. During the experiment, the sea urchins were fed twice a day at 9:00 and 17:00. The feeding amount was equivalent to 5% of the initial body weight of the *S. intermedius*, which was adjusted every week. After each feeding, leftover food and excrement were cleaned up from the plastic box. Throughout the feeding experiment, one-third of the water in all plastic boxes was manually replaced daily with a siphon. The entire feeding period was 114 days. The sea water conditions were as follows: water temperature 20 ± 3°C, salinity 30‰, pH 8.0 ± 0.1 and dissolved oxygen more than 7 mg/L.

### 2.3. Sampling

Upon completion of the feeding experiment, the number, final body weight and final test height and test diameter of the live sea urchins in each tank were measured individually to calculate the survival rate (SR) and weight gain rate (WGR). After that, nine sea urchins were randomly selected from each plastic box, and the body coelomic fluid was extracted from the perioral membrane inserted with a 1 mL syringe and placed into a 1.5-mL enzyme-free centrifuge tube. After centrifugation of the body coelomic fluid of six sea urchins (3000 rpm, 4°C, 5 min), upper liquid was transferred to a new tube, and the samples were nitrogen-frozen in liquid nitrogen and stored at −80°C for later analysis of immune enzyme activities. The body coelomic fluid of three sea urchins was used for phagocytic activity (PA) and respiratory burst analysis. Four sea urchins in each tank were dissected for collecting digestive tracts, which were pooled, nitrogen frozen and stored at −80°C for the analysis of gene expression. The shells of three sea urchins in each plastic box were aseptically dissected and packed, nitrogen-frozen and preserved at −20°C for microbiota analysis.

### 2.4. PA and Respiratory Burst

Sample collection was conducted with minor adjustments following the methodology outlined by Xing et al. [38]. The body coelomic fluid was extracted from the perioral membrane inserted with a 1 mL syringe and placed into a 1.5-mL enzyme-free centrifuge tube. Subsequently, the coelomic fluid was thoroughly blended with an equal volume of anticoagulant solution containing 0.02 M EGTA, 0.48 M NaCl, 0.019 M KCl and 0.068 M Tris-HCl.

The neutral red method was used to measure the phagocytosis activity, with slight modifications based on Zhang et al. [39]. First, 100 μL of anticoagulated coelomic fluid was allowed to stand at 18°C for 30 min to allow the cells in the fluid to adhere to a 1.5 mL centrifuge tube without enzymes. Following the removal of nonadherent cells, 100 μL of neutral red solution (0.001 mol/L) was added to the mixture, which was then gently resuspended and further incubated at 18°C for 45 min. Next, the upper layer of liquid was removed, and sediment was cleaned three times using sterile seawater. Finally, 100 μL of cell lysate with equal amounts of glacial acetic acid and absolute ethanol was added, and the cells were lysed for 20 min. The absorbance value was measured using a wavelength of 540 nm.

The NBT method was employed to measure respiratory burst, with minor adjustments following the approach outlined by Gu et al. [40]. First, 100 μL of anticoagulated coelomic fluid was allowed to stand at 18°C for 30 min to allow the cells in the fluid to adhere to a 1.5 mL centrifuge tube without enzymes. After removing the unadhered cells, 100 μL of NBT solution (1 mg/mL) and 100 μL of (12-) phobotide (-13-) acetate (PMA) were incubated at 18°C for another 45 min. After incubation, the reaction was halted by adding 100 μL of anhydrous methanol. The blue pellet at the bottom of the centrifuge was rinsed three times with 70% methanol and then left to dry at room temperature. Finally, the solution was dissolved in 120 μL of 2 M KOH and 140 μL of dimethyl sulfoxide (DMSO). The absorbance at a wavelength of 630 nm was determined using the KOH/DMSO mixture as the blank solution.

### 2.5. Immune Enzyme Analysis

The samples were processed following the protocol outlined by Li et al. [2]. First, the coelomic fluid was mix well on ice after being mixed 1:9 with phosphate buffered solution. The upper liquid was carefully removed after centrifugation at 4°C (10,000 g) for another 15 min. The protein content was assessed according to Bradford's method. Immune enzyme activities were determined by using commercial kits of total nitric oxide synthase (T-NOS) (AA012-1-2), LYZ (A050-1-1), alkaline phosphatase (AKP) (A059-1-1) and acid phosphatase (ACP) (A060-1-1) (Nanjing Jiancheng Institute, Nanjing, China).

### 2.6. RNA Extraction and Real-Time Quantitative PCR (qPCR)

qPCR was performed with specific primers to determine the expression profiles of genes *LYZ* (JN936415), *TLR* (ADP087831), *TNF* (MH516331), *Caspase-8* [41] *C3* [41] and *APMS* [42] in the intestine of *S. intermedius*. The procedures for RNA extraction and qPCR have been previously described [22]. The RNA from the digestive tract of *S. intermedius* was extracted using Tian Gen's commercially available RNA extraction kit (Tian Gen, Beijing, China). The integrity of the RNA sample was confirmed by agarose gel electrophoresis [43]. The cDNA synthesis procedure involved an initial incubation at 37°C for 15 min, followed by a brief denaturation step at 85°C for 5 s, with subsequent storage of the cDNA at −20°C. cDNA synthesis was performed under the following conditions: incubation at 37°C for 15 min, denaturation at 85°C for 5 s and storage of the cDNA at −20°C. Finally, the first-strand cDNA was diluted fivefold with enzyme-free water. The qPCR was conducted using SYBR Green I chemistry in a 20-μL reaction volume. The reaction mixture comprised 10 μL of 2× TransStart Tip Green qPCR Super Mix, 0.8 μL each of forward and reverse primers (20 μM each), 2 μL of cDNA template and 6.4 μL of RNase-free water. PCR cycling began with an initial denaturation at 95°C for 120 s, followed by 45 cycles of denaturation at 95°C for 5 s and annealing/extension at 95°C for 10 s, concluding with melt curve analysis. Generated standard curves for both the reference and target genes using high-expression samples initially diluted to 50 ng/μL. These samples were then serially diluted tenfold across five points, with each dilution point replicated three times. They identified that the ideal cDNA concentration for the reaction was 5 ng/μL, achieved through a tenfold dilution of the initial 50 ng/μL cDNA solution with DEPC-treated water. The relative expression levels were calculated using the 2^−ΔΔCT^ method. This involved computing ΔCT as the difference between the CT values of the target and reference genes, normalizing ΔCT to a control group to obtain ΔΔCT and determining the fold change as 2^−ΔΔCT^ [44]. The sequences of primers used in this experiment are listed in Table 2.

### 2.7. Microbial Diversity Analysis

The genomic DNA extraction from the bacterial community was carried out by using the E.Z.N.A. DNA Kit (Omega Bio-Tek in Norcross, GA, USA) [46]. The V3–V4 region of the bacterial 16S rRNA was amplified by using an ABI Gene Bad 9700 PCR thermal cycler (ABI, Carlsbad, CA, USA) with the barcoded primers 338F and 806R [47]. The PCR protocol commenced with an initial incubation at 95°C for 3 min, followed by 27 cycles consisting of 30 s at 95°C for denaturation, 30 s at 55°C for annealing and 45 s at 72°C for extension. Finally, there was a 10-min extension step at 72°C. Purification and quantification were performed, followed by merging the amplicons in equimolar amounts after purification and quantification. The amplicons were sequenced on the Illumina MiSeq platform (Illumina, San Diego, CA, USA). The raw reads were filtered based on quality control standards and procedures implemented in QIIME (version 1.17) to obtain high-quality and clean reads [48].

### 2.8. Calculations and Statistical Analysis

  
$$\begin{array}{c} \text{Feed intake }\left(\text{FI},\;\%\right)=\text{consumed food mass}/\left[\left(\text{initial weight + final weight}\right)/2\right]/\text{feeding days}\times 100, \end{array}$$

  
$$\begin{array}{c} \text{Feed conversion ratio }\left(\text{FCR},\;\%\right)=\text{consumed food mass}/\left(\text{final weight − initial weight}\right)\times 100, \end{array}$$

  
$$\begin{array}{c} \text{Survival rate }\left(\text{SR},\;\%\right)=\text{initial numbers of surviving/final numbers of surviving}\times 100, \end{array}$$

  
$$\begin{array}{c} \text{Weight gain rate }\left(\text{WGR},\;\%\right)=\left(\text{final weight − initial weight}\right)/\text{initial weight}\times 100, \end{array}$$

  
$$\begin{array}{c} \text{Gonadosomatic index }\left(\text{GSI},\;\%\right)=\text{final weight gonad/final body weight}\times 100, \end{array}$$

  
$$\begin{array}{c} \text{Test diameter growth }\left(\text{TDG},\;\%\right)=\left(\text{final test diameter − initial test diameter}\right)/\text{initial test diameters}\times 100, \end{array}$$

  
$$\begin{array}{c} \text{Test height growth }\left(\text{THG},\;\%\right)=\left(\text{final test height − initial test height}\right)/\text{initial test height}\times 100. \end{array}$$

SPSS 26.0 software was used for data analysis. The significance of differences among dietary groups was analysed by using one-way ANOVA. When a significant difference (*p* < 0.05) was detected, subsequent multiple comparisons using the Tukey test were performed to evaluate significant variations in mean values across different dietary groups. The findings are presented as the mean ± standard error of the mean (SEM).

## 3. Results

### 3.1. Growth Performance and Feed Utilization

The SR and feed intake (FI) of the sea urchins did not significantly differ among the dietary groups (*p* > 0.05). The WGR of sea urchins significantly increased from 124% to 160% as VA addition level increased from 0 IU/kg to 4000 IU/kg (*p* < 0.05) and then reached a plateau with further increase of VA addition level. The gonadosomatic index (GSI) of sea urchins significantly increased from 13% to 15% as VA addition level increased from 0 IU/kg to 4000 IU/kg (*p* < 0.05) and then reached a plateau with further increase in VA addition level. The test diameter growth rate of sea urchins significantly increased from 45% to 54% as VA addition level increased from 0 to 4000 IU/kg (*p* < 0.05) but decreased with further increase of VA addition level. The test height growth rate of sea urchins significantly increased from 44% to 58% as VA addition level increased from 0 to 4000 IU/kg (*p* < 0.05) but decreased as VA addition level increased further. The feed conversion ratio (FCR) exhibited a contrasting pattern compared to that of the WGR and GSI with increasing VA addition level. The breaking point occurred in the 4000 IU/kg VA addition group (Table 3).

### 3.2. PA and Respiratory Burst

The PA of sea urchins tended to increase as the VA addition level increased from 0 to 32,000 IU/kg but decreased with further increase of VA addition level (*p* < 0.05). However, there were no notable variations in the respiratory burst between the dietary groups (*p* > 0.05) (Table 4).

### 3.3. Immune Enzyme Activities

The activities of T-NOS and ACP showed a notable initial increase followed by a decrease with increasing VA addition level (*p* < 0.05). The peaks occurred in the groups added with 8000 IU/kg and 16,000 IU/kg VA, respectively. The activities of LYZ and AKP showed opposite trends to those of T-NOS and ACP. The lowest value was noted in the group treated with 8000 IU/kg VA (Table 5).

### 3.4. Immune-Related Gene Expression

As the VA addition level increased, several immune-related gene expression of *S. intermedius* first increased and then decreased, with those fed diets with relatively higher addition of VA (32,000 IU/kg) showing significantly upregulated expression of *LYZ*, *TLR*, *TNF*, *Caspase-8* and *AMPs* (*p* < 0.05).

**Figure 1:**
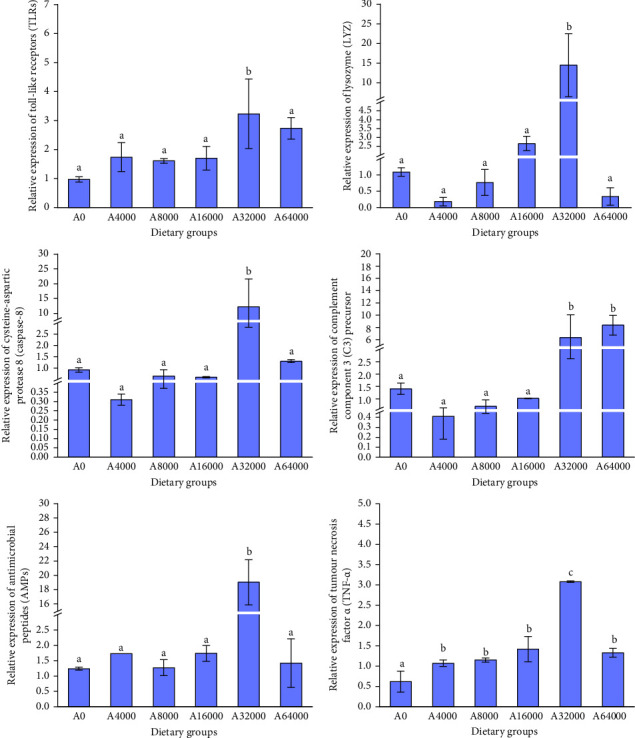
Effects of dietary vitamin A addition level on the relative expression of immune-related genes in the digestive tract of sea urchin (*Strongylocentrotus intermedius*) (mean ± SEM, *n* = 3). Bars labelled with different lowercase letters indicate notable variations (*p*  < 0.05).

The expression of *C3* in the 64,000 IU/mg VA addition level group was notably greater than that in the other dietary groups (*p* < 0.05). There were no notable variations in the expression of *C3* among dietary groups (*p* > 0.05) (Figure 1).

### 3.5. Percent of Sea Urchins With Different Degrees of Erythema

**Figure 2:**
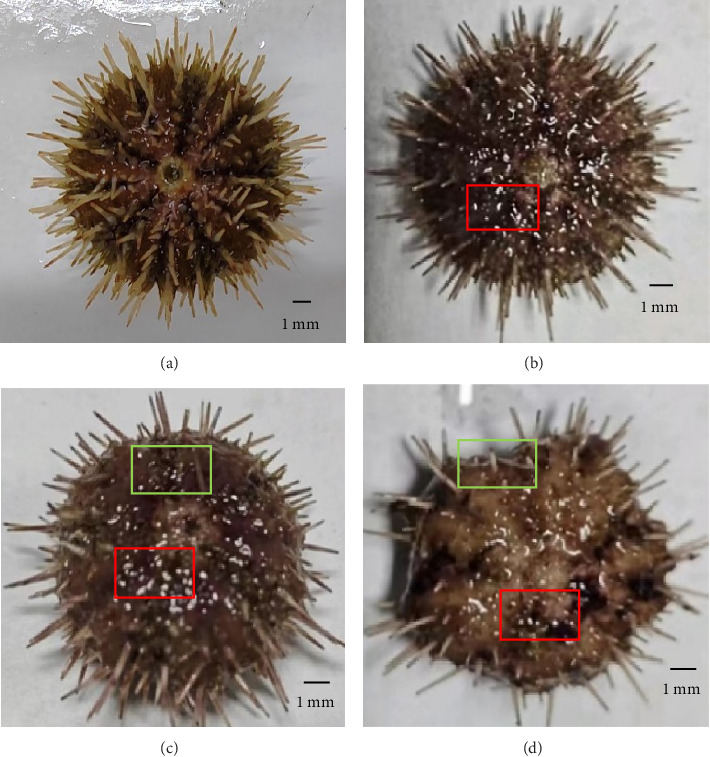
Shell appearance of *Strongylocentrotus intermedius* with different extent of red spotted disease symptoms. (A) Shell appearance of healthy individuals. (B) Shell appearance of mild red spotted disease symptom. (C) Shell appearance of moderate red spotted disease symptom. (D) Shell appearance of severe red spotted disease symptom. Red rectangle: purple spotted lesions or dark red mucus. Green rectangle: broken spines.

Sea urchins with different degrees of erythema have been presented in Figure 2. The percentage of sea urchins with severe red spotted disease decreased from 64.44% to 13.33% as VA addition level increased to 32,000 IU/kg (*p* < 0.05) and subsequently increased to 42.22% with further increase of VA addition level. On the contrary, the percentage of sea urchins with mild red spotted disease increased from 13.33% to 55.55% as VA addition level increased to 32,000 IU/kg (*p* < 0.05) and subsequently decreased to 31.11% with further increase of VA addition level. The percentage of sea urchins with moderate red spotted disease increased from 22.22% to 46.67% as VA addition level increased to 8000 IU/kg (*p* < 0.05) and subsequently decreased to 26.67% with further increase of VA addition level (Table 6).

### 3.6. Shell Microbiome Diversity

The Shannon and Simpson of sea urchins increased significantly as VA addition level increased to 4000 IU/kg (*p* < 0.05). Subsequently, they stabilized despite VA addition level further increased. The expression of ACE and Chao1 was notably increased with VA addition level increasing to 4000 IU/kg and significantly decreased with further increase of VA addition level (*p* < 0.05) (Table 7).

### 3.7. Shell Microbiota Abundance

**Figure 3:**
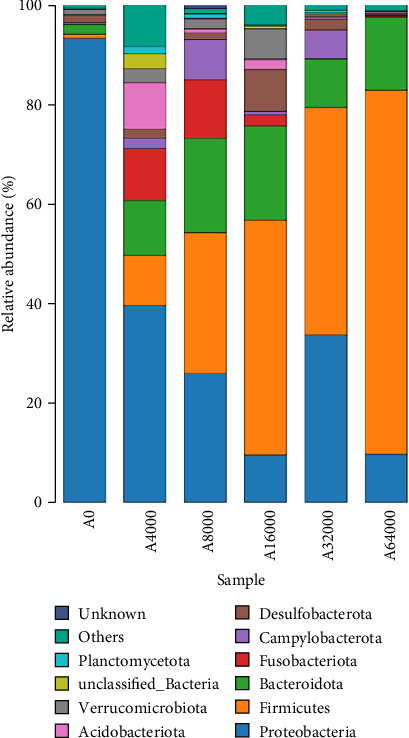
Effects of dietary vitamin A addition levels on the relative abundance of microbiota at the phylum level on the shell surface of juvenile sea urchin (*Strongylocentrotus intermedius*) (standardized to *18S* rRNA) (mean ± SEM, *n* = 3).

At the phylum level, Proteobacteria, Firmicutes and Bacteroidota collectively constituted over 90% of the microbial community that inhabited the shell surface of sea urchins (Figure 3). The abundance of Proteobacteria in sea urchins exhibited an initial decrease followed by an increase as VA addition level rose (*p* < 0.05), reaching their lowest points in the 16,000 IU/kg VA addition group. As the VA addition level increased, there was an initial rise followed by a subsequent decline in the abundance of Bacteroidota (*p* < 0.05). The Bacteroidota abundance was notably higher in the 16,000 IU/kg VA addition group compared to the other feed groups (*p* < 0.05). The abundance of Firmicutes increased significantly with the increase of VA addition level (*p* < 0.05). The abundance of Firmicutes in the 64,000 IU/kg addition group was markedly greater than that in the other groups (Table 8).

**Figure 4:**
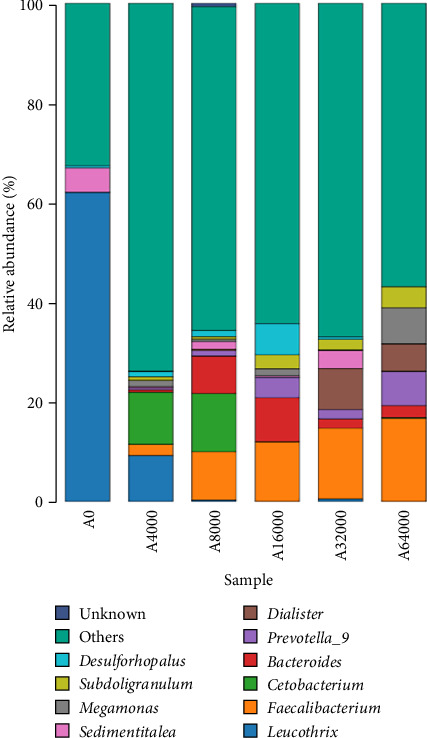
Effects of dietary vitamin A addition levels on the relative abundance of microbiota at the genus level on the shell surface of juvenile sea urchin (*Strongylocentrotus intermedius*) (standardized to *18S* rRNA) (mean ± SEM, *n* = 3).

At the genus level, *Leucothrix*, *Bacteroides* and *Faecalibacterium* were notably influenced by VA addition level (Figure 4). The level of *Leucothrix* in the sea urchin shells decreased notably with increasing VA addition level (*p* < 0.05). The lowest value was noted in the group added with 8000 IU/kg VA. The abundance of *Bacteroides* and *Faecalibacterium* initially rose but then declined as VA addition level increased (*p* < 0.05). The abundance of *Bacteroides* in the 16,000 IU/kg VA addition group was notably greater compared to that in the other feed groups (*p* < 0.05). *Faecalibacterium* in the 32,000 IU/kg VA addition group showed markedly higher relative abundance compared to that in the other feed groups (*p* < 0.05) (Table 9).

## 4. Discussion

VA is essential for supporting the normal growth and physiological functions of aquatic animals [24, 28, 49]. The findings of this research demonstrated that the addition of VA at an appropriate level (4000 IU/kg) significantly promoted the WGR of *S. intermedius*. This value was consistent with the results of Huang et al. [28], who reported that 5340 IU/kg VA resulted in the fastest growth rate of *E. sinensis*. However, these values were greater than those of Liang et al. [50] and Huang et al. [26], who reported that the optimal VA for achieving the highest WGR was 1136 IU/kg for hybrid grouper (*Epinephelus fuscoguttatus* ♀×*Epinephelus lanceolatus*♂) and 2156 IU/kg for *S. sihama*, respectively. On one hand, the different VA requirement could be attributed to variations in animal species, experimental conditions and feed composition. On the other hand, different animals could have different capacity of converting carotenoids to VA [51]. Sea urchins could have a higher requirement for carotenoids to maintain the colour of their gonads and shells, which decreased the percent of carotenoids used for the synthesis of VA. Thus, sea urchins in this experiment showed a higher requirement for VA. Furthermore, as a lipid-soluble vitamin, VA is prone to be easily accumulated and exhibits toxic effects as the feeding period prolonged, which could decrease the estimated VA requirement. In this study, the addition of 64,000 IU/kg VA decreased the WGR of *S. intermedius*. This was consistent with the findings of Mohamed et al. [52] who reported that long-term intake of VA may be toxic to aquatic organisms, reflected by reduced growth rate and immunity. Moren et al. [53] found that long-term intake of high dose of VA disrupted the normal function of cell membranes and organelles including endoplasmic reticulum and mitochondria.

Red spotted disease usually results in high mortality and substantial economic losses [11]. In the present study, the addition of 32,000 IU/kg VA decreased the percentage of *S. intermedius* with severe red spot symptoms (13.33%). Invertebrates are characterized by underdeveloped adaptive immunity and thus rely mainly on innate immunity. Innate immunity is composed of humoral immunity and cellular immunity, mainly phagocytosis and respiratory bursts [54]. As the most abundant cells in the sea urchin coelomic fluid, phagocytes possess the capacity to recognize, capture and destroy foreign particles that enter the body [54]. In the present study, the addition of 32,000 IU/kg VA increased the PA of *S. intermedius*. This was higher than the findings on rats which reported that dietary VA at an addition level of 32,00 IU/kg can significantly increase the PA [55]. ACP is a characteristic lysosomal enzyme within macrophages and is essential for the degradation of foreign substances [56]. In the present study, the addition of 16,000 IU/kg VA increased the ACP activity of *S. intermedius*. Similar findings were reported by Liang et al. [50], who reported that the addition of 7715 IU/kg VA significantly enhanced the ACP activity of juvenile *E. fuscoguttatus♀* × *E. lanceolatus♂*. When phagocytic cells engulf foreign particles, they usually produce large amounts of reactive oxygen species to kill bacteria and microorganisms, which is called a respiratory burst [57]. However, the findings of this study indicated that dietary VA had negligible effects on the respiratory burst of *S. intermedius*. This finding stands in contrast to the results reported by Lian et al. [58], who found that diets containing 3501 IU/kg VA notably boosted respiratory burst activity in renal leukocytes of largemouth bass (*Micropterus salmoides*). This discrepancy could be due to different bacterial killing strategies in different aquatic animals. In the present study, the activities of ACP and LYZ were found to be sensitive to dietary VA. Thus, it was postulated that ACP and LYZ, rather than ROS, play a more important role in killing bacteria inside phagocytes. Huang et al. [28] showed that appropriate VA upregulated *LYZ* expression in juvenile *E. sinensis*. Excessive VA can disrupt lysosomal membranes, leading to the release of hydrolytic enzymes, cell damage and the development of inflammatory responses [59]. In the present study, the addition of 64,000 IU/kg VA to the feed decreased the expression of intestinal ACP and *LYZ* in *S. intermedius*. Liang et al. [50] demonstrated that feeds supplemented with a high level of VA (15,204 IU/kg) decreased the activities of serum ACP, LYZ and *C3*. Furthermore, VA can promote keratinocyte mitosis, increase epithelial thickness and maintain glycosaminoglycans, which can alter the activity of epithelial, melanocytes, fibroblasts and endothelial cells [60]. VA was important for maintaining mucosal and epithelial integrity, increasing collagen and extracellular matrix formation and inducing or maintaining keratinocyte differentiation from the basal layer to the stratum corneum [61]. Thus, the addition of VA at appropriate levels could promote the resistance of *S. intermedius* to red spotted disease by enhancing cellular and humoral immunity and wound repair capacity.

As the VA addition level increased, the abundance of Proteobacteria and *Leucothrix* decreased in this experiment. This was partially supported by Pang et al. [62] and Nan et al. [30], who showed that moderate amounts of VA could reduce the abundance of the intestinal Proteobacteria phylum in *N. vison* and *M. musculus*. The phylum of Proteobacteria includes *Vibrionaceae*, *Sphingosinomonas* and *Rhodomyceae*, which can induce inflammation and cause metabolic disorders [63, 64]. *Leucothrix* has been reported to cause widespread infection of benthic crustaceans and fish eggs as well as high mortality [65]. Thus, the elevated expression of *AMPs* could account for the reduced abundance of *Leucothrix* in the skin of *S. intermedius* fed diets supplemented with 32,000 IU/kg VA. In this study, the abundance of *Faecalibacterium*, Firmicutes, *Bacteroides* and Bacteroidota on the shell surface of sea urchins increased as the VA addition level increased. Martín et al. [66] reported that *Faecalibacterium* improved intestinal homeostasis and prevented colitis in humans. Firmicutes are crucial important for intestinal homeostasis and health, possibly because they utilize the production of dietary polysaccharides and butyrates to improve digestive tract structural integrity, digestion and immunity [67]. *Bacteroides* contribute to maintaining digestive tract homeostasis, and the stability of the body is immune system, as well as providing nutrients and beneficial metabolites to the host or other intestinal commensal bacteria [68]. Wu et al. [69] reported that the administration of 4400 IU/kg VA significantly increased the abundance of *Bacteroides* in the intestine of weaned piglets. The data reported in this study suggest that a relatively high addition of VA may promote the growth of beneficial microflora and reduce the amount of harmful microflora on the shell surface of *S. intermedius*. This could explain the beneficial effects of relatively higher VA addition on the inhibition of severe red spot disease in *S. intermedius*.

*TLRs* are among the earliest and most widely studied pattern recognition receptors (PRRs) in invertebrates and are involved in innate immune defence against invasion by exogenous pathogens [70]. Upon pathogen recognition, the toll/IL-1 receptor (*TIR*) domain of *TLRs* triggers downstream signalling, which leads to the activation of nuclear factor-kB and promotes the transcription of genes for inflammatory cytokine [71]. Increasing *TLR* expression can improve the PA of apoptotic virus-infected cells [72]. *TNF-α* is a type of inflammatory cytokine that is involved in a series of processes, such as apoptosis, cell proliferation and pathogen prophylaxis [73]. In this study, the addition of 32,000 IU/kg VA increased the expression of *TLR* and *TNF* in *S. intermedius*. Jiang et al. [74] showed that supplementation with 2000 IU/kg VA in the diet significantly increased *TLR* gene expression in the hepatopancreas of *E. sinensis*. The results of this investigation suggest that relatively higher addition of VA can enhance the nonspecific immunity of *S. intermedius*, which is partially attributed to the upregulating effects of VA on the expression of immune genes. In this study, the addition of 32,000 IU/kg VA increased the expression of *AMPs* in *S. intermedius*. Liang et al. [50] showed that diets supplemented with 4142 IU/kg VA significantly increased *AMP* gene expression in *E. fuscoguttatus* ♀ × *E. lanceolatus* ♂. *AMPs* can directly kill harmful microorganisms on the skin, including bacteria, viruses, fungi and parasites [75]. *AMPs* can inhibit the colonization of *Staphylococcus aureus* and thereby reduce secondary skin infections and persistent inflammation [76].

## 5. Conclusions

In conclusion, the best growth performance of *S. intermedius* was observed when the addition level of VA was equal to or above 4000 IU/kg dry feed. However, relatively high levels of VA (32,000 IU/kg) achieved the best nonspecific immunity and red spotted disease resistance. The expression of immune-related genes was inhibited by excess or insufficient VA in dry feeds. The relative abundance of Firmicutes, Bacteroidota and *Faecalibacterium* was increased, while that of Proteobacteria and *Leucothrix* on the shell surface of *S. intermedius* was reduced by increasing dietary VA. These results suggested that a low addition level of VA (4000 IU/kg) can help *S. intermedius* achieve ideal growth performance. However, relatively higher addition levels of VA (32,000 IU/kg) enhanced nonspecific immunity and red spotted disease resistance of *S. intermedius*, which could be accomplished by promoting immune gene expression and optimizing the shell microbiota composition.

## Figures and Tables

**Table 1:** Formulation and approximate composition of the experimental diets (% dry weight).

Ingredients (%)	Dietary groups					
	A0	A4000	A8000	A16000	A32000	A64000
Fish meal^a^	0	4	4	4	4	4
Soybean meal^b^	10	10	10	10	10	4
Wheat meal^c^	10	10	10	10	10	10
Corn starch^d^	10	10	10	10	10	10
Gluten^e^	5	5	5	5	5	5
Seaweed powder^f^	20	20	20	20	20	20
Wheat bran^g^	32.7	32.7	32.7	32.7	32.7	32.7
Vitamin A	0	0.0004	0.0008	0.0016	0.0032	0.0064
Mineral premix	2.00	2.00	2.00	2.00	2.00	2.00
Multivitamins	2.00	2.00	2.00	2.00	2.00	2.00
Calcium propionate	0.18	0.18	0.18	0.18	0.18	0.18
Ethoxyquin	0.01	0.01	0.01	0.01	0.01	0.01
Choline chloride	0.10	0.10	0.10	0.10	0.10	0.10
Fish oil	2.40	2.40	2.40	2.40	2.40	2.40
Soybean lecithin	1.60	1.60	1.60	1.60	1.60	1.60
Proximate analysis						
Crude protein (%)	20.15	20.17	20.08	20.18	20.11	20.14
Crude lipid (%)	6.10	6.07	6.13	6.09	6.11	6.05

*Note:* Mineral premix (mg or g kg^−1^ diet): CuSO_4_ · 5H_2_O, 10 mg; Na_2_SeO_3_ (1%), 25 mg; ZnSO_4_ · H_2_O, 50 mg; CoCl_2_ · 6H_2_O (1%), 50 mg; MnSO_4_ · H_2_O, 60 mg; FeSO_4_ · H_2_O, 80 mg; Ca(IO_3_)_2_, 180 mg; MgSO_4_ · 7H_2_O, 1200 mg; zeolite, 18.35 g. Vitamin premix (mg or g/kg diet): vitamin D, 5 mg; vitamin K, 10 mg; vitamin B_12_, 10 mg; vitamin B_6_, 20 mg; folic acid, 20 mg; vitamin B_1_, 25 mg; vitamin B_2_, 45 mg; pantothenic acid, 60 mg; biotin, 60 mg; niacin, 200 mg; vitamin E, 240 mg; inositol, 800 mg; vitamin C, 2000 mg; and microcrystalline cellulose, 16.505 g.

^a^Fish meal: crude protein 68.7%, crude lipid 9.7%.

^b^Soybean meal: crude protein 51.5%, crude lipid 0.9%.

^c^Wheat meal: crude protein 13.8%, crude lipid 1.0%.

^d^Cornstarch: crude protein 0.3%, crude lipid 2.0%.

^e^Gluten: crude protein 80%, crude lipid 2.8%.

^f^Seaweed powder: crude protein 8.15%.

^g^Wheat bran: crude protein 16.4%, crude lipid 4.0%.

**Table 2:** Real-time quantitative PCR primers used in the present study.

Gene	Sequence (5′–3′)	Reference
18S	F: GTTCGAAGGCGATCAGATAC	Ding et al. [45]
	R: CTGTCAATCCTCACTGTGTC	
LYZ	F: GAGACGGTACAGGGCTACA	JN936415
	R: CGGGCAAAATCCTCACAAG	
C3	F: AGAATGGTGCTGTGAGGGTGC	Zhang et al. [42]
	R: CCGTGATACTACTTGGAGTGGAGA	
Caspase-8	F: CCCTCTCTTGTGGGCAAACC	Zhang et al. [42]
	R: TGCATGACTCATCCGCTCGT	
TLR	F: ATCTGAAACTGCTTCCAAACGAC	ADP087831
	R: AGAGAGGGCTTGGATTTCGTT	
TNF-*α*	F: GCTGTAACGGCGTTCGTCTCC	MH516331
	R: TGGTGTACTTGTGCTGGTTGTTGG	
AMPs	R: GGATCTCAGGAGCGCATCC	Zhou et al. [41]
	F: TAGGTTGATGCCCGGCATA	

Abbreviations: AMPs, antimicrobial peptides; C3, complement component 3 precursor; Caspase-8, cysteine-aspartic protease 8; LYZ, lysozyme; TLR, toll-like receptors; TNF-*α*, tumour necrosis factor *α*.

**Table 3:** Effects of dietary vitamin A addition levels on the growth performance of juvenile sea urchin (*Strongylocentrotus intermedius*) (means ± SEM, *n* = 3).

Index	Dietary groups					
	A0	A4000	A8000	A16000	A32000	A64000
Survival rate (SR, %)	96.30 ± 6.41	100.00 ± 0.00	98.13 ± 3.23	94.77 ± 5.06	96.267 ± 3.23	92.90 ± 2.60
Weight gain rate (WGR, %)	124.66 ± 26.06^a^	160.87 ± 33.43^b^	164.31 ± 39.81^b^	166.94 ± 29.39^b^	173.62 ± 40.24^b^	159.96 ± 57.06^b^
Test diameter growth (TDG, %)	45.96 ± 17.03^a^	54.75 ± 14.14^b^	50.01 ± 15.47^a,b^	51.68 ± 15.25^a,b^	50.64 ± 16.44^a,b^	47.19 ± 20.36^a^
Test height growth (THG, %)	43.98 ± 18.17^a^	58.55 ± 14.96^c^	51.56 ± 16.21^b,c^	51.67 ± 15.24^b,c^	51.29 ± 16.94^b,c^	49.10 ± 21.55^a,b^
Gonadosomatic index (GSI, %)	13.10 ± 3.22^a^	15.82 ± 4.65^b^	15.80 ± 4.05^b^	15.67 ± 4.18^b^	16.32 ± 3.97^b^	14.80 ± 4.53^a,b^
Feed intake (FI, %)	4.52 ± 0.24	4.31 ± 0.34	4.57 ± 0.49	4.80 ± 0.12	4.55 ± 0.12	4.74 ± 0.35
Feed conversion ratio (FCR, %)	2.33 ± 0.32^a^	2.01 ± 0.25^a^	2.17 ± 0.24^a^	2.18 ± 0.03^a^	2.48 ± 0.19^b^	3.03 ± 0.67^b^

*Note:* Data with distinct lowercase letters within the same row are notably different (*p* < 0.05).

**Table 4:** Effects of dietary vitamin A addition levels on the phagocytic activity and respiratory burst of juvenile sea urchin (*Strongylocentrotus intermedius*) (means ± SEM, *n* = 3).

Index	Dietary groups					
	A0	A4000	A8000	A16000	A32000	A64000
Phagocytic activity (OD550/10^6^ cells)	1.01 ± 0.029^a^	1.04 ± 0.26^a^	1.12 ± 0.55^a^	1.25 ± 0.29^a,b^	1.72 ± 0.46^b^	1.41 ± 0.36^a,b^
Respiratory burst (OD630/10^6^ cells)	0.09 ± 0.10	0.10 ± 0.04	0.10 ± 0.04	0.09 ± 0.03	0.25 ± 0.30	0.11 ± 0.03

*Note:* Data with distinct lowercase letters within the same row are notably different (*p* < 0.05).

**Table 5:** Effects of dietary vitamin A addition levels on the immune enzyme activities of juvenile sea urchin (*Strongylocentrotus intermedius*) (means ± SEM, *n* = 3).

Index	Dietary groups					
	A0	A4000	A8000	A16000	A32000	A64000
T-NOS (U/mL)	27.38 ± 4.06^a^	31.10 ± 2.40^a,b^	33.84 ± 8.38^b^	28.61 ± 3.97^a,b^	28.23 ± 0.86^a,b^	28.78 ± 1.87^a,b^
LYZ (U/mL)	28.40 ± 5.68^c^	20.37 ± 2.14^a,b^	18.52 ± 3.70^a^	23.46 ± 2.14^a,b,c^	23.46 ± 2.14^a,b,c^	25.93 ± 4.28^b,c^
AKP (King unit/100 mL)	1.55 ± 0.42^a,b,c^	1.29 ± 0.30^a,b^	1.21 ± 0.21^a^	1.61 ± 0.12^b,c^	1.80 ± 0.50^a,b,c^	1.85 ± 0.12^c^
ACP (King unit/100 mL)	1.67 ± 0.38^a^	1.84 ± 0.40^a,b^	2.12 ± 0.28^a,b^	2.42 ± 0.41^b^	2.37 ± 0.86^b^	1.81 ± 0.46^a,b^

*Note:* Data with distinct lowercase letters within the same row are notably different (*p* < 0.05).

Abbreviations: ACP, acid phosphatase; AKP, alkaline phosphatase; LYZ, lysozyme; T-NOS, total nitric oxide synthase.

**Table 6:** Effects of dietary vitamin A addition levels on the prevalence of different degrees of red spotted disease of juvenile sea urchin (*Strongylocentrotus intermedius*) (mean ± SEM, *n* = 3).

Index	Dietary groups					
	A0	A4000	A8000	A16000	A32000	A64000
Mild red spot disease (%)	13.33 ± 6.67^a^	22.22 ± 3.85^b^	31.11 ± 3.85^c^	37.78 ± 3.85^c^	55.55 ± 3.85^d^	31.11 ± 3.85^c^
Moderate red spot disease (%)	22.22 ± 13.88^a^	31.11 ± 3.85^a,b^	46.67 ± 6.67^c^	40.00 ± 6.67^b,c^	31.11 ± 3.85^a,b^	26.67 ± 6.67^a,b^
Severe red spot disease (%)	64.44 ± 19.25^c^	48.89 ± 3.85^b^	22.22 ± 10.18^a^	22.22 ± 3.85^a^	13.33 ± 6.67^a^	42.22 ± 3.85^a,b^

*Note:* Data with distinct lowercase letters within the same row are notably different (*p* < 0.05).

**Table 7:** Effects of dietary vitamin A addition levels on microbial diversity in shell of juvenile sea urchin (*Strongylocentrotus intermedius*) (mean ± SEM, *n* = 3).

Index	Dietary groups					
	A0	A4000	A8000	A16000	A32000	A64000
Shannon	3.29 ± 0.52^a^	7.03 ± 0.63^c^	5.22 ± 1.49^b^	5.73 ± 0.09^a,b^	5.42 ± 0.23^b^	5.30 ± 0.50^b^
Simpson	0.62 ± 0.11^a^	0.96 ± 0.02^b^	0.91 ± 0.06^b^	0.94 ± 0.01^b^	0.94 ± 0.01^b^	0.94 ± 0.01^b^
ACE	550.76 ± 206.85^a,b^	771.22 ± 72.45^b^	393.10 ± 42.31^a^	529.88 ± 84.71^a,b^	383.59 ± 97.81^a^	345.51 ± 26.43^a^
Chao1	467.45 ± 88.13^a^	807.61 ± 98.49^b^	423.50 ± 2.12^a^	519.27 ± 122.63^a^	359.78 ± 36.46^a^	326.93 ± 40.42^a^

*Note:* Data with distinct lowercase letters in the same row are significantly different (*p* < 0.05).

**Table 8:** Effects of dietary vitamin A addition levels on the relative abundance of microbiota at the phylum level on the shell surface of juvenile sea urchin (*Strongylocentrotus intermedius*) (mean ± SEM, *n* = 3).

Phylum	Dietary groups					
	A0	A4000	A8000	A16000	A32000	A64000
Proteobacteria	0.93 ± 0.00^c^	0.41 ± 0.16^b^	0.26 ± 0.16^a,b^	0.11 ± 0.07^a^	0.14 ± 0.0.08^a^	0.04 ± 0.03^a^
Firmicutes	0.01 ± 0.00^a^	0.04 ± 0.02^a,b^	0.1 ± 0.02^b^	0.22 ± 0.03^c^	0.70 ± 0.02^d^	0.75 ± 0.05^d^
Bacteroidota	0.02 ± 0.00^a^	0.11 ± 0.02^a,b^	0.19 ± 0.09^b^	0.20 ± 0.05^b^	0.09 ± 0.07^a,b^	0.15 ± 0.09^b^

*Note:* Data with distinct lowercase letters in the same row are significantly different (*p* < 0.05).

**Table 9:** Effects of dietary vitamin A addition levels on the relative abundance of microbiota at the genus level on the shell surface of juvenile sea urchin (*Strongylocentrotus intermedius*) (mean ± SEM, *n* = 3).

Genus	Dietary groups					
	A0	A4000	A8000	A16000	A32000	A64000
*Leucothrix*	0.64 ± 0.10^c^	0.15 ± 0.05^b^	0.00 ± 0.00^a^	0.00 ± 0.00^a^	0.00 ± 0.00^a^	0.00 ± 0.00^a^
*Bacteroides*	0.00 ± 0.00^a^	0.01 ± 0.00^a^	0.11 ± 0.04^b^	0.15 ± 0.06^b^	0.03 ± 0.01^a^	0.03 ± 0.01^a^
*Faecalibacterium*	0.00 ± 0.00^a^	0.00 ± 0.00^a^	0.00 ± 0.00^a^	0.00 ± 0.00^a^	0.23 ± 0.10^b^	0.18 ± 0.01^b^

*Note:* Data with distinct lowercase letters within the same row are notably different (*p* < 0.05).

## Data Availability

The data of this study can be provided by the corresponding author upon request.
